# *In silico *microarray probe design for diagnosis of multiple pathogens

**DOI:** 10.1186/1471-2164-9-496

**Published:** 2008-10-21

**Authors:** Ravi Vijaya Satya, Nela Zavaljevski, Kamal Kumar, Elizabeth Bode, Susana Padilla, Leonard Wasieloski, Jeanne Geyer, Jaques Reifman

**Affiliations:** 1 Biotechnology HPC Software Applications Institute, Telemedicine and Advanced Technology Research Center, U.S. Army Medical Research and Materiel Command, Fort Detrick, MD 21702, USA; 2 Diagnostic Systems Division, US Army Medical Research Institute of Infectious Diseases, Fort Detrick, MD 21702, USA

## Abstract

**Background:**

With multiple strains of various pathogens being sequenced, it is necessary to develop high-throughput methods that can simultaneously process multiple bacterial or viral genomes to find common fingerprints as well as fingerprints that are unique to each individual genome. We present algorithmic enhancements to an existing single-genome pipeline that allows for efficient design of microarray probes common to groups of target genomes. The enhanced pipeline takes advantage of the similarities in the input genomes to narrow the search to short, nonredundant regions of the target genomes and, thereby, significantly reduces the computation time. The pipeline also computes a three-state hybridization matrix, which gives the expected hybridization of each probe with each target.

**Results:**

Design of microarray probes for eight pathogenic *Burkholderia *genomes shows that the multiple-genome pipeline is nearly four-times faster than the single-genome pipeline for this application. The probes designed for these eight genomes were experimentally tested with one non-target and three target genomes. Hybridization experiments show that less than 10% of the designed probes cross hybridize with non-targets. Also, more than 65% of the probes designed to identify all *Burkholderia mallei *and *B. pseudomallei *strains successfully hybridize with a *B. pseudomallei *strain not used for probe design.

**Conclusion:**

The savings in runtime suggest that the enhanced pipeline can be used to design fingerprints for tens or even hundreds of related genomes in a single run. Hybridization results with an unsequenced *B. pseudomallei *strain indicate that the designed probes might be useful in identifying unsequenced strains of *B. mallei *and *B. pseudomallei*.

## Background

Sequence-based pathogen identification is an increasingly important tool for clinical diagnostics and environmental monitoring of biological threat agents. Developments in sequencing technology have led to the availability of many pathogen genome sequences. Many more pathogen genomes and near-neighbors are being sequenced due to initiatives by the National Institute of Allergy and Infectious Diseases and the U.S. Department of Defense. Availability of these genomic sequences has opened up opportunities for the development of whole-genome-based diagnostic assays, such as DNA microarrays and polymerase chain reaction (PCR) assays, which offer more flexibility than traditional methods based on a single gene or selected regions of a target genome [1]. Microarray-based pathogen diagnostic assays are gaining popularity due to their ability to test for hundreds, or even thousands, of pathogens in a single diagnostic test [2].

Oligonucleotide probes designed for pathogen diagnostic assays should be unique to the pathogen with respect to all other non-target genomes. Clinical and environmental samples may contain a multitude of non-target genomes, and hence probes designed for diagnostic assays must be unique with respect to all non-target genomes. As a result, the design of pathogen diagnostic assays entails the computationally expensive comparison of target genomes with all known non-target sequences. Many different methods have been developed to guide the design of pathogen diagnostic assays. Some methods [3-5] are intended for PCR-based assays, whereas others [6-12] are intended for microarray-based assays. Kaderali and Schliep [6] presented one of the first methods for designing microarrays for pathogen identification. Their approach is very similar to that of designing probes for gene expression analysis; they design a single probe for each target, with the probe being unique to the target with respect to all other input target sequences. However, the specificity of the probe with respect to other non-target genomes is not analyzed. A somewhat similar approach is that of *host-blind *probe design presented by Putonti *et al*. [13], in which the probes are unique only with respect to a host genome. With a few exceptions, however, most of these tools do not have the capability of testing for specificity against a large number of non-target genomes. This is clearly not adequate if the signatures are to be used to identify the pathogen from environmental/clinical samples containing any number of unanticipated non-target organisms.

Some tools for designing PCR assays, such as KPATH [5] and Insignia [4], perform *in silico *comparisons against all known non-target sequences. They also have the ability to design common signatures for multiple pathogen sequences. In KPATH, common PCR signatures are selected from a multiple sequence alignment of the target genomes. As described by Fitch *et al*. [3], this approach is inherently based on the assumption of collinearity within the target genomes, which may not hold true for bacterial genomes. Insignia, on the other hand, selects common signatures from shared sequences discovered through pairwise local alignments and, hence, does not assume collinearity.

Neither KPATH nor Insignia is applicable for designing microarray fingerprints, as the design and specificity requirements of microarray fingerprints are quite different from those of PCR signatures. The most commonly used PCR signatures consist of a probe and two primers and, due to their short length [18–25 base pairs (bp)] and constraints on the interprimer distance, inexact matches with non-target sequences can be tolerated without much degradation in specificity. Conversely, in addition to being characterized by only one DNA segment with no spacing constraints, microarray probes are generally longer and more susceptible to cross hybridization even in the absence of an exact match [14]. This requires more extensive searches, for both exact and inexact matches, against non-target sequences to identify highly specific fingerprints for microarrays.

We have previously developed a software tool [9,12] for designing microarray probes that identify fingerprints for a single target genome. The software, named TOFI (Tool for Oligonucleotide Fingerprint Identification), is an integrated, scalable, high-performance-computing pipeline, which combines genome comparison tools, probe design software, and sequence alignment programs to design highly specific microarray probes for pathogen identification. To our knowledge, TOFI is the only software that has the ability to design microarray probes that are specific to the target with respect to all sequenced non-target genomes.

In this paper, we extend the TOFI pipeline to design microarray probes for multiple, related, bacterial and viral pathogens. Our aim is to efficiently identify all probes in the input target sequences that are unique with respect to all available non-target sequences. The major contributions of this paper include: (1) an efficient algorithm that pre-processes the input target sequences to take advantage of the similarities among them and reduce their effective size, which results in considerable speedup (nearly four-fold for eight targets and greater for larger numbers) when compared to the previous, single-target version of the pipeline, (2) a novel three-state hybridization matrix computation strategy, which identifies probes that can be used to characterize the possible combinations of the input target genomes, and (3) a set of experimental results with multiple *Burkholderia *genomes, which allow us to validate the new algorithms and the specificity criteria that were recently introduced (but not experimentally validated) in [12]. The improved pipeline scales well with increase in the number of target genomes, and can potentially design fingerprints for hundreds of related target genomes in a single run.

## Methods

**Figure 1:**
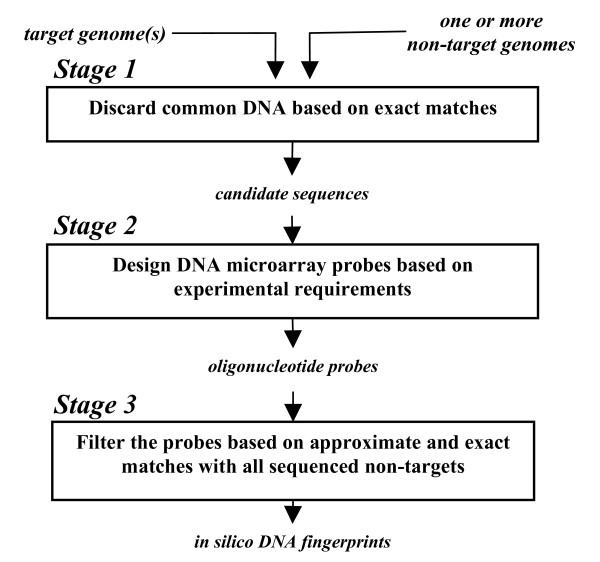
**Overview of the TOFI pipeline**. Stage 1 and Stage 3 of the TOFI pipeline have been improved to handle multiple genomes. In stage 1, the target genomes are compared with each other to eliminate redundant sequences. In Stage3, an *in silico *hybridization matrix is computed, which indicates which probes hybridize to which targets.

In the following, we briefly describe the TOFI pipeline for a single genome, and then present the algorithmic improvements implemented to accommodate multiple genomes. The TOFI pipeline consists of the three main stages illustrated in Figure 1. The stages are designed so that large portions of the target genome are eliminated in the less-expensive two initial stages, and the computationally more expensive searches for specific fingerprints are performed over smaller regions of the target genome in the final stage. The reader should refer to [12] for a detailed description of the TOFI pipeline.

### Overview of TOFI pipeline

The first stage of TOFI uses the suffix-tree-based MUMmer [15] program to perform pairwise comparisons of the target genome with each non-target genome and eliminate regions in the target genome that have exact matches with any of the non-target genomes. Given a pair of sequences, MUMmer finds all maximal matches that are at least as long as a threshold (termed *minmatch*) between the two sequences. TOFI uses MUMmer to find these maximal matches and eliminate regions in the target genome that are covered by them. The selection of *minmatch *is based on the specificity parameters supplied by the user. This ensures that every segment of the target genome that satisfies the restrictions on probe length and specificity parameters is part of the surviving regions of the target genome. These surviving regions, referred to as candidate sequences, are then passed on to the second stage of the pipeline.

In the second stage, TOFI identifies oligonucleotides of desired length from the candidate sequences that satisfy experimental conditions, such as melting temperature (*T*_*m*_) and GC content. TOFI uses the Oligonucleotide Modeling Platform (OMP) software to identify these oligonucleotides, also referred to here as probes. OMP uses the nearest-neighbor hybridization model [16] to calculate *T*_*m *_and to estimate if a probe forms any secondary structures that may prevent it from hybridizing to the intended target.

In the third and final stage of the pipeline, TOFI performs a BLAST [17] search for each probe against a comprehensive sequence database, such as the *nt *database provided by the National Center for Biotechnology Information (NCBI). The BLAST comparisons are performed in parallel on multiple processors using the blastn program of mpiBLAST [18]. Probes with significant alignments to non-target genomes are eliminated, and the surviving probes become the *in silico *DNA fingerprints for the target genomes. These probes are then subjected to experimental validation to test their sensitivity and specificity.

### Multiple Genomes

Given a set of target genomes, our aim is to find microarray fingerprints that are unique to any subset of the target genomes with respect to all sequenced non-target genomes. The input consists of a set of *K *target genomes *T *= {*t*_1_, *t*_2_, ..., *t*_*K*_}, where each target *t*_*k*_, 1 ≤ *k *≤ *K*, is a collection of all the sequences (a FASTA file containing chromosomes, unassembled contigs, etc.) from the *k*th target genome. The aim is to select a set of *N *probes *P *= {*p*_1_, *p*_2_, ..., *p*_*N*_}, where each probe *p*_*n *_of length |*p*_*n*_| is a substring of some sequence in *T*, with *L*_*min *_≤ |*p*_*n*_| ≤ *L*_*max*_, and *L*_*min *_and *L*_*max *_representing the minimum and maximum probe length constraints, respectively. In addition, a probe *p*_*n *_should satisfy experimental constraints like GC content and melting temperature, and should not have significant sequence similarity with any known genomic sequence not in *T*.

The multiple-genome pipeline differs from the single-genome pipeline in Stage 1 and Stage 3. The major enhancements include: (1) comparison of each target genome with all other target sequences to eliminate redundant sequence segments from further consideration in Stage 1, and (2) computation of an *in silico *hybridization matrix of patterns in Stage 3, where each pattern identifies the input target sequences that can be characterized by a probe.

### Preprocessing the target genomes

A brute-force approach for designing fingerprints for multiple genomes would entail the design of fingerprints for each genome separately. For *K *target genomes, this approach would take approximately *K *times the computation time necessary for designing fingerprints for a single genome. Given the recent sequence availability of multiple bacterial strains of interest (ranging from tens to hundreds) and the large computation time to identify fingerprints for a typical bacterial genome (~5 hours on 74 processors), such brute-force approach would be impractical.

In general, there is significant sequence similarity among closely related genomes. Our approach takes maximum advantage of such similarities by eliminating (redundant) sequence segments that are shared by any two input target genomes. This is done as the first step in Stage 1, where TOFI compares the targets within themselves to construct a set of nonredundant target sequences. The fingerprints for the multiple targets are then designed from these nonredundant target sequences. Comparison of the input target sequences is performed using an iterative process that effectively compares each target genome with all other target genomes.

We start with the set of target genomes *T *and a set of nonredundant target sequences *S*, which is initially empty. All sequences in the first target genome *t*_1 _are added to *S*. Next, we find all exact matches between the sequences in *t*_2 _and *S *using MUMmer. All exact matches that are longer than an input threshold are removed from *t*_2_, and the remaining sequences are added to *S*. This process is sequentially repeated for all other target genomes. When processing the *k*th genome, all nonredundant sequences from the previous *k*-1 sequences are already included in *S*. Hence, only the nonredundant sequences in *t*_*k *_are added to *S*. In this process, *minmatch*, the threshold for minimum exact matches, should be equal to *L*_*min*_. If *minmatch *is larger than *L*_*min*_, shared sequences of length ≥*L*_*min *_not reported by MUMmer will be included in *S*, leading to highly similar segments being added to the list of nonredundant sequences. The nonredundant sequences at the end of this preprocessing step are then subjected to comparisons with non-target sequences to remove exact matches with non-target sequences. The candidate sequences at the end of Stage 1 are submitted to Stage 2, which selects probes satisfying the experimental constraints. These probes are then subjected to extensive specificity analysis in Stage 3, which includes hierarchical BLAST comparisons against increasingly larger databases of non-target sequences [12].

### *In silico *Hybridization Expectations

As the probes are designed to identify multiple target genomes, any given probe is not necessarily a substring of every target. As a result, it is necessary to explicitly compare each probe against each target sequence to identify the targets for which the probe can serve as a fingerprint. We consider multiple criteria in determining the specificity of a probe. Many measures, such as overall sequence identity, contiguous matches, and predicted free energy, have all been previously shown to be important measures of the potential for cross hybridization [19,20]. In addition to these measures, we use several measures of near-contiguous matches introduced in a previous paper on TOFI [12]. To incorporate contiguous and near-contiguous matches in determining probe specificity and estimate hybridization expectations, we use a series of thresholds, *M*_0_, *M*_1_, *M*_2_, and *M*_3_, where *M*_*i *_is the maximum length of a contiguous region in which the alignment between a probe and a genome sequence has (*M*_*i *_- *i*) matches and *i *mismatches/insertions/deletions. Accordingly, *M*_0 _is the length of the longest stretch of contiguous matches between a probe and a genome sequence. Identity, contiguous matches, and near-contiguous matches between a probe and a target/non-target genome are computed from the BLAST alignments between the two.

We use two sets of thresholds to compute *in silico *hybridization expectations. The first set of design thresholds, denoted by *C*^*U*^, indicates the minimum value of each parameter necessary for hybridization. The second set of thresholds, denoted by *C*^*L*^, indicates the maximum value of each parameter permissible for avoiding hybridization. *C*^*U *^is used to identify probes that will potentially hybridize to a genome, and *C*^*L *^is used to identify probes that will not hybridize to a genome. The individual thresholds in *C*^*U *^are denoted by *Identity*^*U*^, *M*_0_^*U*^, *M*_1_^*U*^, *M*_2_^*U*^, and *M*_3_^*U*^, and the individual thresholds in *C*^*L *^are denoted by *Identity*^*L*^, *M*_0_^*L*^, *M*_1_^*L*^, *M*_2_^*L*^, and *M*_3_^*L*^. For a probe to be considered as an *in silico *fingerprint for a target, all specificity measures between the probe and the target sequence must be greater than the corresponding thresholds in *C*^*U *^and all specificity measures between the probe and any non-target sequence must be less than or equal to the corresponding thresholds in *C*^*L*^. Note that the set of thresholds *C*^*U *^is only employed to compute the *in silico *hybridization expectations, which are used to identify the targets for which each probe can serve as a fingerprint. Accordingly, the number of probes reported by TOFI is solely controlled by the thresholds in *C*^*L*^.

We use the pairwise BLAST program bl2seq to compare each probe with each target sequence. Based on the alignments of the *N *probes with the *K *targets, we build an *N *× *K *hybridization matrix *H*, where each entry *H*_*nk *_indicates whether probe *p*_*n *_hybridizes to target *t*_*k*_. Unlike earlier representations [21-23], which use a binary matrix to represent the hybridization expectations, we use a three-state matrix. Each *H*_*nk *_∈ {-1,0,1}, where *H*_*nk *_= 1 indicates that fingerprint *p*_*n *_hybridizes with genome *t*_*k*_, *H*_*nk *_= -1 indicates that *p*_*n *_does not hybridize to *t*_*k*_, and *H*_*nk *_= 0 indicates that *p*_*n *_may or may not hybridize to *t*_*k*_. We opt for a three-state representation to more accurately represent the expected behavior of the probes. In many situations, probes may have some sequence similarity with a given target, but this identity may not be high enough to guarantee hybridization or low enough to rule out the possibility of hybridization.

The hybridization matrix is constructed based on the highest scoring alignment between a probe *p*_*n *_and a target genome *t*_*k*_, as follows:

i. *H*_*nk *_= -1 if all the specificity measures are less than or equal to the corresponding thresholds in *C*^*L*^;

ii. *H*_*nk *_= 1 if all the specificity measures are greater than the corresponding thresholds in *C*^*U*^; and

iii. *H*_*nk *_= 0 if neither (i) nor (ii) is satisfied.

A probe *p*_*n *_is considered unique to a target *t*_*k *_if *H*_*nk *_= 1, and *H*_*nj *_= -1 ∀ *j *≠ *k*. A probe *p*_*n *_is common to a set of targets *T*_*s *_if:

i. *H*_*nj *_= 1 ∀ *j *| *t*_*j *_∈ *T*_*s*_, and

ii. *H*_*nj *_= -1 ∀ *j *| *t*_*j *_∉ *T*_*s*_.

According to this definition, any probe *p*_*n *_with *H*_*nk *_= 0 for any target *k *can neither be a probe unique to a target nor a probe common to a set of targets. However, as these are only *in silico *expectations, some of these probes may prove to be useful after experimental validation.

## Results

In this section, we present the results for the identification of *in silico *fingerprints (i.e., probe design) and their associated experimental evaluation. For the probe design process, we used probe length parameters *L*_*min *_= 35 and *L*_*max *_= 40, and optimal melting temperature of 70°C. Probe lengths of 35–40 bases were chosen to ensure compatibility of the probe sequences with microarrays available from various vendors, some of which are limited to the *in situ *synthesis of probes that are 40 bases or less. We used the entire NCBI *nt *database to estimate probe specificity.

### Probe design

We designed probes for four strains of *Burkholderia mallei *and four strains of *B. pseudomallei*, employing *B. thailandensis *as the non-target near-neighbor genome. Table 1 shows the details of the eight target genomes and the near-neighbor genome, each consisting of two chromosomes. The table shows the combined sizes of the two chromosomes.

**Table 1:** NCBI accession numbers and sizes of the *Burkholderia *genomes used for probe design

	Strain	Accession no./version	Size (bp)
1	*B. pseudomallei *1106a	NC_009076.1, NC_009078.1	7089249
2	*B. pseudomallei *1710b	NC_007434.1, NC_007435.1	7308054
3	*B. pseudomallei *668	NC_009074.1, NC_009075.1	7040403
4	*B. pseudomallei *K96243	NC_006350.1, NC_006351.1	7247547
5	*B. mallei *ATCC 23344	NC_006348.1, NC_006349.1	5835527
6	*B. mallei *NCTC 10229	NC_008835.1, NC_008836.1	5742303
7	*B. mallei *NCTC 10247	NC_009079.1, NC_009080.1	5848380
8	*B. mallei *SAVP1	NC_008784.1, NC_008785.1	5232401
9	*B. thailandensis *E264 (near-neighbor)	NC_007651.1, NC_007650.1	6723972

As expected, there is significant sequence similarity among the eight genomes. The four strains of *B. pseudomallei *are significantly different from each other. As a result, the combined nonredundant sequence size increased as each new *B. pseudomallei *sequence was processed. Conversely, the four strains of *B. mallei *are very similar to each other and to the four *B. pseudomallei *genomes, therefore only minimally increasing the combined nonredundant size as these genomes were processed. The combined size of the target genomes is 51343862 bp. However, the combined size of all nonredundant target sequences after the preprocessing step in Stage 1 was just 12011005 bp, a reduction of more than 75%. These nonredundant target sequences were further compared against the entire *nt *database retrieved from NCBI in July 2007. This version of the *nt *database consists of more than 5 million sequences with combined size greater than 21 Gbp.

The strategy of identifying and eliminating redundant portions of the target sequences considerably reduced the overall computation time. The total time to design fingerprints for the eight *Burkholderia *genomes with TOFI on a 74-processor Linux cluster with distributed memory was 9 hours and 41 minutes. Using the same number of processors, it took approximately 4 hours and 30 minutes to design fingerprints for each genome, for a total of 36 hours for processing the eight genomes. Therefore, for the eight *Burkholderia *genomes tested, the strategy used in the multiple-genome pipeline yielded a nearly four-fold reduction in the computation time in comparison with the single-genome pipeline. The savings in computation time would be even greater with larger number of target genomes.

Table 2 shows the values for the specificity thresholds *C*^*L *^and *C*^*U *^used for specificity computation with non-targets and targets, respectively. The thresholds for *M*_0_^*L *^and *Identity*^*L *^were selected based on those suggested in the literature [14,19,24], and making the necessary adjustments to obtain a reasonably large number of fingerprints. The relaxation of these thresholds and the selection of other thresholds in *C*^*L *^and *C*^*U *^were based on empirical analyses of free energy computations previously presented [12]. Based on the *C*^*L *^thresholds, a total of 5015 probes were expected to be free of cross hybridization with non-targets. Table 3 shows the number of probes expected to identify each target. The third column in the table shows the number of probes that passed the design thresholds *C*^*U *^for each target strain; meaning that these are the number of probes that should hybridize with each strain. The fourth column indicates the number of probes that are unique to each target; meaning that these probes have matches ≤*C*^*L *^with all other genomes, including the other seven target strains. Column five shows the number of probes common to each subgroup, and the last column shows the number of probes common to all eight target strains.

**Table 2:** The specificity thresholds used for probe design

*C*^*L*^	*C*^*U*^
*Identity*^*L *^= 85%	*Identity*^*U *^= 90%
*M*_0_^*L *^= 18	*M*_0_^*U *^= 27
*M*_1_^*L *^= 21	*M*_1_^*U *^= 29
*M*_2_^*L *^= 24	*M*_2_^*U *^= 32
*M*_3_^*L *^= 27	*M*_3_^*U *^= 34

**Table 3:** Expected behavior of the 5015 designed probes

	Target	Total	Unique	Group	Common
1	*B. pseudomallei *1106a	2710	259	504	981
2	*B. pseudomallei *1710b	3346	739	504	981
3	*B. pseudomallei *668	2597	601	504	981
4	*B. pseudomallei *K96243	3084	613	504	981
5	*B. mallei *ATCC 23344	1373	0	31	981
6	*B. mallei *NCTC 10229	1339	0	31	981
7	*B. mallei *NCTC 10247	1567	0	31	981
8	*B*. *mallei *SAVP1	1164	0	31	981

In all, 981 probes out of the total 5015 are expected to identify all eight strains. A total of 504 probes are unique to the *B. pseudomallei *subgroup, meaning that these probes have matches >*C*^*U *^with all the four *B. pseudomallei *strains and matches ≤*C*^*L *^with all other organisms, including the four *B. mallei *strains. Similarly, a total of 31 probes are unique to the *B. mallei *subgroup. There are hundreds of unique probes for each individual *B. pseudomallei *strain. However, because of the high similarity between the *B. mallei *genomes, none of the 5015 probes are unique to any individual *B. mallei *strain.

### Hybridization experiments

Efficient hybridization of bacterial DNA requires that long genomic DNA molecules be fragmented to shorter lengths for optimal hybridization. In our experiments, we used restriction endonuclease digestion to fragment bacterial DNA prior to labeling and hybridization. Therefore, all probes that overlapped with restriction site positions corresponding to restriction enzymes used to prepare the DNA for hybridization were not included. This reduced the number of probes from 5015 to 2343. To reduce the number of probes further, we prioritized probes based on their predicted hybridization patterns and selected a total of 1214 probes by eliminating probes that were neither unique to an individual strain nor common to all strains in any of the two *Burkholderia *species. We conducted hybridization experiments on a total of 1817 probes, which include an additional set of 603 probes. Most of these 603 probes were selected based on sub-optimal specificity thresholds to assess the effect of various specificity criteria on cross hybridization. These 603 probes included 79 probes that were duplicated for verifying consistency of hybridization intensities with individual probes.

Samples from five different strains, *B. mallei *ATCC 23344, *B. mallei *NCTC 10229, *B. pseudomallei *K96243, *B. pseudomallei *238, and *B. thailandensis *E264, were prepared for hybridization by restriction digestion in a cocktail of five restriction endonucleases: Rsa I, Hpa II, Hinp1 I, HpyCH4 IV, and Alu I (New England Biolabs, Ipswich, MA). Fragmented DNA was labeled with a ULYSIS 647 Nucleic Acid Labeling Kit (Invitrogen, Carlsbad, CA), following the manufacturer's instructions. One microgram of each labeled DNA was hybridized onto one segment each of three replicate custom Agilent 8x15K Comparative Genomic Hybridization (CGH) microarray chips (Agilent Technologies, Santa Clara, CA), following the manufacturer's instructions. Each CGH microarray chip consisted of eight identical individual array segments, each with 15000 features. Each individual array segment contained seven replicates each of the 1817 selected *Burkholderia *probes and seven replicates each of 31 negative control probes. The remaining unspecified features were populated by Agilent control probes, which were not included in the analysis. Hybridized chips were scanned with a GenePix 4000B Axon Scanner, using GenePix Pro 6.1 software (Molecular Devices, Sunnyvale, CA) at 5 μm for individual spot evaluation.

### Evaluation of experimental results

We analyzed the hybridization results using microarray data analysis functions available in the MATLAB Bioinformatics Toolbox . We normalized the data among corresponding arrays in each of the three chips using the quantile normalization method [25] and logarithmically transformed the normalized hybridization intensities. Subsequently, we subtracted the background, which was estimated for each array on each chip using the set of 31 negative control probes. The resulting normalized values were used for evaluating the probes. The complete list of 1817 probes and their normalized hybridization intensities are given in Additional file 1.

Table 4 shows the median (*m*_*B*_) and standard deviation (*σ*) of the estimated non-logarithmically transformed background intensities. The background intensities were very consistent across the three chips (not shown), as well as among the three *Burkholderia *strains. Similar to probe design, we used two empirical thresholds *R*^*L *^and *R*^*U *^to classify probes based on these normalized hybridization data. We selected the lower threshold *R*^*L *^to assess cross hybridization with non-targets to be slightly less than 3*σ *above the background (see Table 4), *R*^*L *^= 0.5. For the upper threshold *R*^*U*^, which is used to assess hybridization with the intended targets, we selected a very conservative value of more than 6*σ *above the background, *R*^*U *^= 1.0.

**Table 4:** Background hybridization intensities averaged over three chips for three *Burkholderia *genomes

	*B. pseudomallei *K96243	*B. mallei *ATCC 23344	*B. mallei *NCTC 10229
Median background intensity (*m*_*B*_)	3710	3076	3890
Standard deviation of background intensity (σ)	548	463	569
$\log_{2}\left(\frac{m_{B}+3\sigma }{m_{B}}\right)\approx R^{L}$	0.53	0.54	0.53
$\log_{2}\left(\frac{m_{B}+6\sigma }{m_{B}}\right)\approx R^{U}$	0.92	0.92	0.90

To enable consistent comparison between probes designed by TOFI and the experimental results, we re-evaluated the number of *in silico *fingerprints in a manner that simulates the experimental setup, using the same design thresholds *C*^*L *^and *C*^*U *^indicated in Table 2. Table 5 illustrates the different combinations of the three target strains, *B. mallei *ATCC 23344, *B. mallei *NCTC 10229, and *B. pseudomallei *K96243, that were available for experimental analysis. The first column denotes the five different groups of probes or categories we compared, and the second column identifies the number of re-evaluated *in silico *(i.e., design) probes. Categories I, II and III correspond to probes unique to individual targets, while treating all other genomes as non-targets. Probes in category IV are common to both *B. mallei *ATCC 23344 and *B. mallei *NCTC 10229, considering all other genomes (including *B. pseudomallei *K96243) as non-targets. Probes in Category V are common to all three target genomes, considering *B. thailandensis *as the non-target.

**Table 5:** Evaluation of *in silico *(design) probes against hybridization results with *R*^*U *^= 1.0 and *R*^*L *^= 0.5 for five categories of probes

Category (targets)	*In silico*	Experimental		
		
		Class A	Class B	Class C
I (*B. mallei *ATCC 23344)	0	0 (0%)	0 (0%)	0 (0%)
II (*B. mallei *NCTC 10229)	2	0 (0%)	2 (100%)	0 (0%)
III (*B. pseudomallei *K96243)	523	420 (80%)	53 (10%)	50 (10%)
IV (*B. mallei *ATCC 23344 and *B. mallei *NCTC 10229)	21	17 (81%)	2 (10%)	2 (10%)
V (*B. mallei *ATCC 23344, *B. mallei *NCTC 10229 and *B. pseudomallei *K96243)	431	184 (43%)	12 (3%)	236 (55%)

Probes that behave as expected are categorized as Class A; i.e., these probes have normalized hybridization intensity greater than *R*^*U *^with intended targets and less than *R*^*L *^with non-targets. Class B probes have normalized hybridization intensity greater than *R*^*L *^with non-targets, and Class C probes have normalized hybridization intensity less than *R*^*U *^with the intended targets.

Based on the experimental results, we classify the probes in each category into three classes (Table 5). Class A corresponds to probes that have normalized hybridization intensity greater than *R*^*U *^with the intended targets and normalized hybridization intensity less than or equal to *R*^*L *^with non-targets. Class B designates probes that have normalized hybridization intensity greater than *R*^*L *^with non-targets, whereas Class C designates probes that have normalized hybridization intensity less than or equal to *R*^*U *^with the intended targets. Therefore, Class A probes are the probes that behave as expected. Note that some probes can be in both Class B and Class C, and that the purpose of the design criteria *C*^*U *^and *C*^*L *^is to maximize the number of probes in Class A and minimize the number of probes in Class B and Class C.

Due to the high similarity between the two *B. mallei *genomes, the probes in Categories I and II (both *in silico *and experimental) are too few for analysis. According to the design criteria, 523 probes out of the 1817 are unique to *B. pseudomallei *(Category III). According to the experimental thresholds, 420 (80%) of these are in Class A, meaning that they hybridize with *B. pseudomallei *and do not hybridize with any of the other genomes. Relatively few probes in this category are in Class B or Class C. The probes unique to both strains of *B. mallei *(Category IV) perform similarly. A large fraction (81%) of these probes is in Class A. The probes in Category V, which are expected to hybridize with all three target genomes, behaved differently. Less than half of these probes (43%) are in Class A, whereas the majority (55%) of these probes are in Class C, meaning that they are failing to hybridize with some or all of the intended targets.

### Performance against an unsequenced target

The robustness of common probes designed to identify a group of targets can be evaluated by testing their hybridization against another member of the group that was not included in the design process. Accordingly, we obtained the hybridization results of the 1817 probes with *B. pseudomallei *238, for which the genome sequence is not available from NCBI. Hybridization results with this strain might provide insights into how common probes designed based on a limited set of strains of *B. pseudomallei *and *B. mallei *would perform on unsequenced strains of these pathogens. Table 6 shows the performance of these group-specific probes as the result of hybridization experiments with *B. pseudomallei *238. The 302 *in silico *probes in Category VI represent the subset of the 1817 probes that are expected to identify all eight target genomes listed in Table 1. Similarly, the 92 probes in Category VII are expected to identify all four *B. pseudomallei *genomes in Table 1.

**Table 6:** Experimental hybridization results of group-specific *in silico *probes tested against *B. pseudomallei *238

Category (targets)	*In silico*	Probes hybridizing with *B. pseudomallei *238
VI (*B. mallei *and *B. pseudomallei*)	302	236 (78%)
VII (*B. pseudomallei *specific)	92	60 (65%)

Out of the 302 probes in Category VI, 236 (78%) have normalized hybridization intensity greater than *R*^*U *^= 1.0 with *B. pseudomallei *238 and less than *R*^*L *^= 0.5 with *B. thailandensis *E264. Similarly, out of the 92 *B. pseudomallei*-specific probes in Category VII, 60 (65%) probes have normalized hybridization intensity greater than *R*^*U *^with *B. pseudomallei *238, and less than *R*^*L *^with the two strains of *B. mallei *and the strain of *B. thailandensis *tested. These results indicate that these group-specific probes can be used to identify *B. pseudomallei *238, and possibly other unsequenced strains of *B. mallei *and *B. pseudomallei*.

### Experimental inconsistencies

Based on the two design thresholds, *C*^*U *^and *C*^*L*^, all 431 probes in Category V are expected to hybridize to all three targets. However, as shown in Table 5, only 184 of these are in Class A, whereas many (236) are in Class C. To understand why this might be happening, we looked at the number of probes out of these 431 that hybridize to each of the three individual targets. We found that 343 probes (80%) hybridized with *B. mallei *ATCC 23344, 408 probes (95%) hybridized with *B. mallei *NCTC 10229, and only 196 probes (45%) hybridized with *B. pseudomallei *K96243. This clearly indicates that most of the probes in category V failed to hybridize with *B. pseudomallei *K96243.

**Figure 2:**
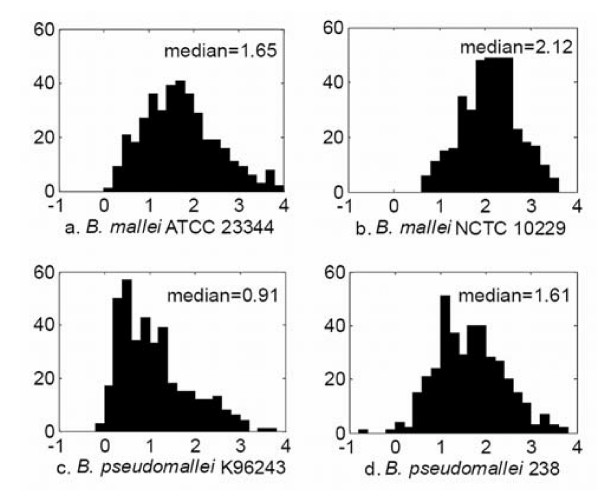
**Histograms of normalized hybridization intensities for the 382 probes that have 100% identity with the three target genomes**. The X-axis shows the normalized hybridization intensities and the Y-axis shows the number of probes that have a given normalized hybridization intensity. Many of the 382 probes fail to hybridize with *B. pseudomallei *K96243 even though all these probes have 100% identity with this genome, whereas hybridization intensities for the other three genomes are as expected.

Further insights can be gained by looking at the probes that have 100% identity with each of the three targets. There are 382 such probes, and all of them are expected to hybridize to all three targets. Figure 2 shows the histograms of the normalized hybridization intensities for these 382 probes. Figures 2a, 2b, and 2c indicate that hybridization intensities with the *B. mallei *strains are significantly higher than those with *B. pseudomallei *K96243. The median hybridization intensities for both strains of *B. mallei *are substantially above the experimental threshold *R*^*U *^= 1.0, which is not the case for *B. pseudomallei *K96243. Hybridization with the unsequenced strain *B. pseudomallei *238 in Figure 2d is also comparable to that of the two *B. mallei *strains, with a median value (1.61) higher than the threshold (1.0), indicating that a large fraction of these 382 probes can be used to detect this strain. Hybridization with *B. thailandensis *E264 (not shown) is at the background level.

Based on these histograms, one might be tempted to conclude that the hybridization intensities with *B. pseudomallei *K96243 are lower than those for the remaining strains due to some experimental anomaly. However, this does not seem to be the case because most of the 523 probes in category III (Table 5) that are expected to hybridize only to *B. pseudomallei *K96243 perform as expected. The median hybridization for these 523 probes is 2.44, which is well above the threshold 1.0. Currently, we do not have an explanation for why the strain-specific probes are hybridizing as expected with *B. pseudomallei *K96243, whereas group-specific probes are failing. We are investigating the causes for the observed discrepancies.

## Conclusion

The enhanced TOFI pipeline can efficiently design microarray fingerprints for multiple, related bacterial and viral genomes. We designed probes for eight pathogenic *Burkholderia *genomes, covering probes unique to single targets as well as probes common to groups of targets. Probe design results show that the presented method is effective in taking advantage of the commonalities among the genomes to considerably reduce the overall computation time (about a four-fold reduction in this case, with larger gains for larger number of input targets). This indicates that the pipeline can be used to design fingerprints for a large number of related microbial genomes in a single run. In addition, the computational efficiency of the pipeline allows quick reevaluation of the probes as new target/non-target sequences become available.

This study also allowed us to assess and experimentally validate new specificity criteria recently introduced by Vijaya Satya et al. [12]. Preliminary hybridization results, with three targets, one unsequenced target, and one non-target, demonstrate that only a small percentage of the designed probes (≤10%) cross hybridize with non-targets (last three rows of Class B in Table 5). However, additional tests with a larger panel of non-target genomes are necessary to qualify the selected probes for diagnostic assays. More than 65% of the group-specific probes identify the unsequenced *B. pseudomallei *238 strain, which suggests that these probes might be useful in identifying new strains of *B. mallei *or *B. pseudomallei*.

## Authors' contributions

RVS conceived and implemented the algorithmic enhancements and KK upgraded the user interface for processing multiple genomes. LW designed and guided the experiments. EB and SP conducted the hybridization experiments. JG provided overall guidance for the hybridization experiments. NZ analyzed the hybridization data. JR provided overall project guidance for the computational efforts. RVS, NZ, LW and JR were the main writers of the manuscript. All authors reviewed the manuscript.

## Availability and requirements

• **Project name: **TOFI

• **Project home page: **

• **Operating systems: **Linux

• **Programming Language: **Perl

• **Other Requirements: **mpiBLAST 1.4.0 or higher, MUMmer 3.19 or higher, and OMP developer edition

## Supplementary Material

Additional file 1 **List of probes and their normalized hybridization intensities**. The file contains the list of 1817 probes that were tested experimentally. The first three columns give the probe name, probe sequence, and probe length, respectively. Columns 4 through 8 provide the average normalized hybridization intensity values for *B. mallei *ATCC 23344, *B. mallei *NCTC 10229, *B. pseudomallei *K96243, *B. pseudomallei *238, and *B. thailandensis *E264, respectively. The averages were computed across three chips, where each probe was replicated seven times on each chip. Click here for file
